# Polyacrylic-Coated Solid Nanoparticles Increase the Aquaporin Permeability to Hydrogen Peroxide

**DOI:** 10.3390/ijms25010372

**Published:** 2023-12-27

**Authors:** Giorgia Pellavio, Maria Paola Demichelis, Patrizia Sommi, Umberto Anselmi-Tamburini, Claudia Scotti, Umberto Laforenza

**Affiliations:** 1 Human Physiology Unit, Department of Molecular Medicine, University of Pavia, 27100 Pavia, Italy; giorgia.pellavio@unipv.it (G.P.); patrizia.sommi@unipv.it (P.S.); 2 Department of Chemistry, University of Pavia, 27100 Pavia, Italy; mariapaola.demichelis01@universitadipavia.it (M.P.D.); tau@unipv.it (U.A.-T.); 3 Unit of Immunology and General Pathology, Department of Molecular Medicine, University of Pavia, 27100 Pavia, Italy; claudia.scotti@unipv.it; 4 Center for Health Technologies (CHT), University of Pavia, 27100 Pavia, Italy

**Keywords:** HeLa, oxidative stress, water channels, peroxiporin, HyPer7 biosensor, CeO_2_NP, Gd_2_O_3_NP, Fe_3_O_4_NP, TiO_2_NP

## Abstract

Aquaporins (AQPs) allow the diffusion of hydrogen peroxide (H_2_O_2_) and act as ROS scavenging systems, which are important for controlling the redox state of cells. Recently, cerium oxide nanoparticles were found to increase the water and H_2_O_2_ permeability by modulating AQPs. To further analyze the action of nanoparticles (NPs) on AQP, we examined the effect of the NPs presenting different core compositions (CeO_2_, Gd_2_O_3_, Fe_3_O_4_, and TiO_2_), hydrodynamic sizes, and surface functionalization. The NPs produced an increase in H_2_O and H_2_O_2_ permeability as a general trend. The hydrodynamic sizes of the NPs in the range of 22–100 nm did not produce any significant effect. The chemical nature of the NPs’ core did not modify the effect and its intensity. On the other hand, the NPs’ functionalized surface plays a major role in influencing both water and H_2_O_2_ permeability. The results suggest that NPs can play a significant role in controlling oxidative stress in cells and might represent an innovative approach in the treatment of a number of pathologies associated with an increased oxidative status.

## 1. Introduction

Aquaporins (AQPs) are water channel proteins that facilitate the diffusion of water and some small solutes [1,2]. An increasing number of paralogs (AQP0, 1, 3, 5, 6, 8, 9, and AQP11) mediate the diffusion of hydrogen peroxide (H_2_O_2_) and represent the sub-family of the peroxiporins [3,4,5,6,7,8,9,10,11,12,13,14]. The peroxiporins’ permeability to H_2_O_2_ has been found to be reduced by different stress conditions [3,15] through a sort of negative feedback. The negative effect of high levels of H_2_O_2_ on its permeability to AQP can worsen cellular oxidative stress and lead to cell death [3,10]. Recently, it has been observed that, in contrast to this behavior, mesothelioma-immortalized cells showed increased AQP-mediated permeability to water and H_2_O_2_ in response to oxidative stress through positive feedback, making these cells resistant to apoptosis [14]. 

Hydrogen peroxide is the principal actor responsible for the oxidative stress condition since it is one of the most abundant and stable reactive oxygen species (ROS) in organisms. For this reason, H_2_O_2_ elimination is a key mechanism for maintaining H_2_O_2_ at a physiological concentration at which it acts as a second messenger. As a result, reductions in the intracellular level of H_2_O_2_ participate in reducing the overall ROS levels. In promoting H_2_O_2_ elimination from the cell, AQPs represent an important antioxidant system and potential targets for innovative treatments of pathologic conditions such as cancer and degenerative diseases [16]. Different compounds, such as small molecules, heavy metal ion inhibitors, and antibodies have been screened to identify substances capable of modulating their permeability [15,16,17,18,19,20].

More recently, we studied the effect of cerium oxide nanoparticles or ceria (CeNPs) on the AQP-mediated permeability to water and H_2_O_2_ for their antioxidant activity [21]. CeNPs were found to increase both permeabilities by interacting with AQP8 and to a lesser extent with AQP3 and AQP6. Moreover, CeNPs were much more effective in increasing the permeability of AQPs in oxidative stress conditions. So far, CeNPs are the first compounds capable of increasing the efficiency of AQPs. Thus, nanoparticles represent a new class of AQP modulators with promising drug features that might potentially be useful in diseases caused by high levels of free radicals. 

Although our study has clarified one antioxidant target of CeNPs [21], several questions remain unsolved about the mechanism underlying the interaction of CeNPs with AQPs. Three main features could determine the activity of CeNPs on AQPs: (1) the chemical nature of the core, (2) the hydrodynamic size, and (3) the surface functionalization. In the case of CeNPs, the core was represented by cerium oxide in which Cerium can present two oxidation states, Ce(III) and Ce(IV). The dynamic equilibrium between these two states would generate an oxidation-reduction cycle, making CeNP a self-regenerating oxidant scavenger. The surface of the CeNPs was functionalized with polyacrylic acid (PAA), which has a very strong affinity towards cell membranes [22]. Functionalization is important to stabilize the NPs’ suspension in the solvent, preventing their aggregation. Finally, CeNPs were small nanoparticles with a diameter of about 14 nm. The nanoparticles’ size can influence the amount taken up by endocytosis and can determine the final localization [23,24].

Herein, we investigated the above-mentioned variables that may be responsible for the activity of nanoparticles on AQP-mediated H_2_O_2_ permeability. The elimination of hydrogen peroxide is one of the most effective methods to reduce the cellular oxidative state [10] and thus could be considered one of the antioxidant systems of the cell. HeLa cells were used because they are well characterized in relation to AQP expression and function and were already used in our previous studies analyzing the effects of CeNPs [3,15,21,22].

To this aim, we have studied the effect of different nanoparticles on AQP-mediated water and H_2_O_2_ permeability. In particular, we examined the effect of nanoparticles with (1) different core compositions (iron oxide, FeNPs, gadolinium oxide, GdNPs, and titanium oxide, TiNPs); (2) different hydrodynamic dimensions (NPs small (S-NP), with a diameter below 15 nm, and NPs large (L-NP), with a diameter of about 40–80 nm); and (3) surface functionalization (PAA and dextran (dex)). 

The results reported here provide evidence that, regardless of the core component, small NPs with a PAA surface functionalization were the most effective in increasing the AQP-mediated permeability to H_2_O_2,_ in addition to being characterized by low toxicity. These results are the first indication that NPs with specific chemical-physical characteristics could influence AQP activity. In this respect, the NPs’ antioxidant activity exerted by potentiating the detoxifying action of AQP could be considered for the development of innovative treatments for diseases with increased oxidative status such as degenerative disorders.

## 2. Results

The effect of different NPs on AQP was evaluated by measuring water and H_2_O_2_ permeability. The NPs used for this study were synthesized considering the following characteristics: NPs’ core composition, hydrodynamic size, and surface functionalization. 

### 2.1. Nanoparticles Characterization 

**Figure 1:**
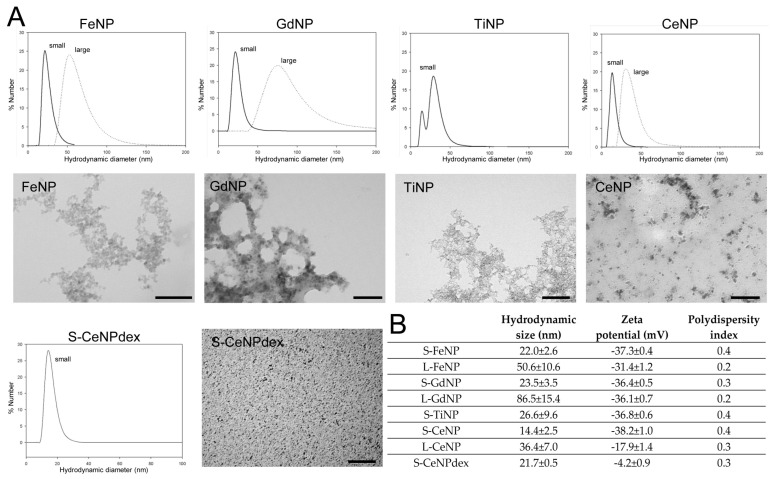
(**A**) Hydrodynamic size graph (distribution by number) of both small and large FeNPs, GdNPs, TiNPs, and CeNPs and dextran-coated CeNPs (S-CeNPdexs) with the corresponding TEM images. Note that the aggregate aspect of the crystals visible in the TEM images is the result of the sample preparation and does not reflect the real aggregation state in the original suspension. Bars, 100 nm. (**B**) Summary of hydrodynamic size, zeta potential, and polydispersity index values for all nanoparticles.

The produced nanoparticles were characterized by their hydrodynamic size and surface charge. NPs were stable in aqueous suspension for about a month. NP hydrodynamic size distributions are shown in Figure 1A, and results are summarized in Figure 1B.

Characterization through Transmission Electron Microscopy (TEM) was also carried out. Figure 1A shows that the nanoparticles, although presenting a hydrodynamic size between 14 and 86 nm, were composed of smaller crystals. PAA-coated FeNPs presented the largest crystal size, about 7 nm, whereas PAA-coated CeNPs presented crystals ranging from 4 to 6 nm, the same as GdNP, and TiNPs consisted of grains with sizes between 3 and 5 nm.

### 2.2. Cellular Viability

**Figure 2:**
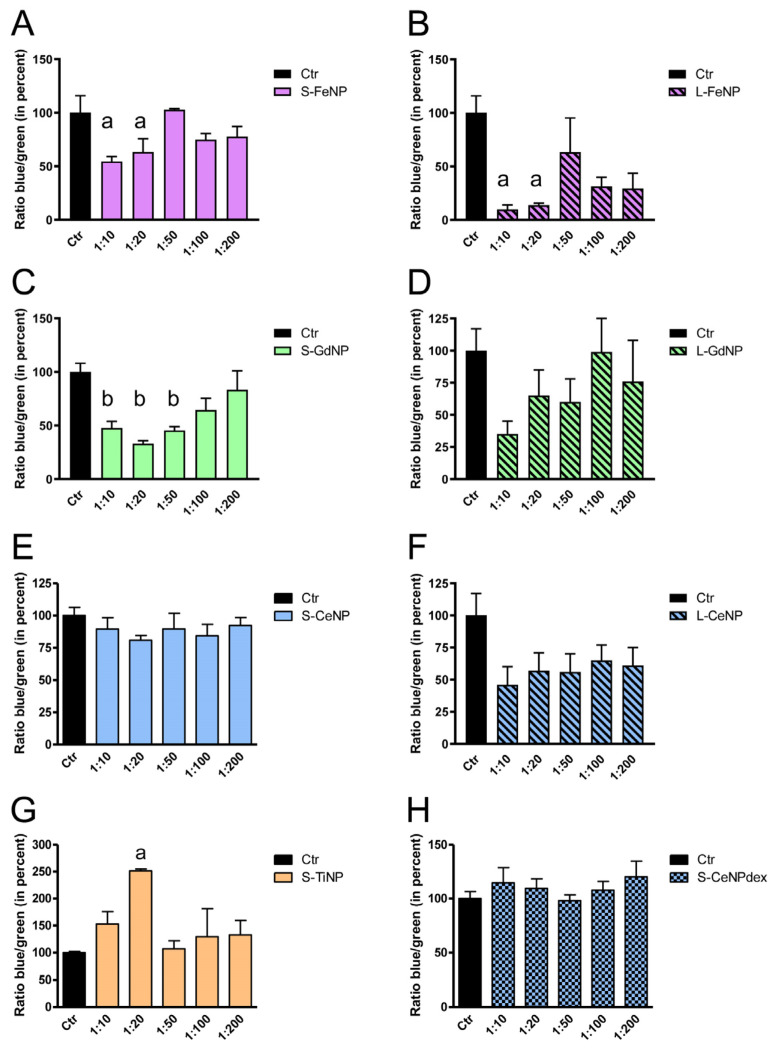
Effect of S-FeNPs (**A**), L-FeNPs (**B**), S-GdNPs (**C**), L-GdNPs (**D**), S-CeNPs (**E**), L-CeNPs (**F**), S-TiNPs (**G**), and S-CeNPdexs (**H**) on the cell viability of HeLa. Cells were incubated for 2 h with nanoparticles diluted 1:10, 1:20, 1:50, 1:100, and 1:200 in PBS. Cell viability was calculated by measuring the blue (live cells; Ex 360nm–Em 460 nm) over the green (dead cells; Ex 420 nm–Em 535 nm) fluorescence signal ratio in treated cells versus untreated cells (Ctr). Ratio values expressed in percentage (normalized to the total protein content) are the mean ± S.E.M. of cells for each of the three different experiments. a, *p* < 0.05 versus Ctr; b, *p* < 0.001 versus Ctr (ANOVA, followed by Dunnett’s multiple comparison test). Concentration of the NPs’ stock solutions: S-FeNP and L-FeNP, 5.4 mg/mL; S-GdNP and L-GdNP, 6.4 mg/mL; S-CeNP (**E**) and L-CeNP, 6.6 mg/mL; S-TiNP, 6.0 mg/mL; S-CeNPdex, 6.5 mg/mL.

We first tested cell viability upon NP exposure to find the right NP concentrations to be used for the investigation of the AQP functional experiments. For each type of NP, HeLa cells were treated with different dilutions (from 1:10 to 1:200) and compared with untreated cells. In the case of FeNPs, the dilution that did not modify the cell viability was 1:50 for both S-FeNPs and L-FeNPs. Higher dilutions, like 1:10 and 1:20, significantly decrease the number of viable cells (Figure 2A,B) with a reduction of 46% and 37% or 90% and 86% for S- and L-FeNPs, respectively. For the GdNPs, 1:100 was the dilution of choice for both S-GdNPs and L-GdNPs (Figure 2C,D). S-CeNPs did not affect cell viability, at least in the range studied (from 1:10 to 1:200) (Figure 2E). For L-CeNPs, 1:100 was also the dilution of choice since all the different dilutions tested did not show a decrease in cell viability (Figure 2F). In the case of TiNPs, the vitality was not altered by any of the dilutions tested (Figure 2F). Interestingly, at 1:20 dilution, an increment in the number of viable cells was observed, making it the dilution of choice for the following functional experiments. Finally, the S-CeNPdex did not modify the cell viability (Figure 2H).

### 2.3. The Effect of Nanoparticles on HeLa Cells Hydrogen Peroxide Permeability

**Figure 3:**
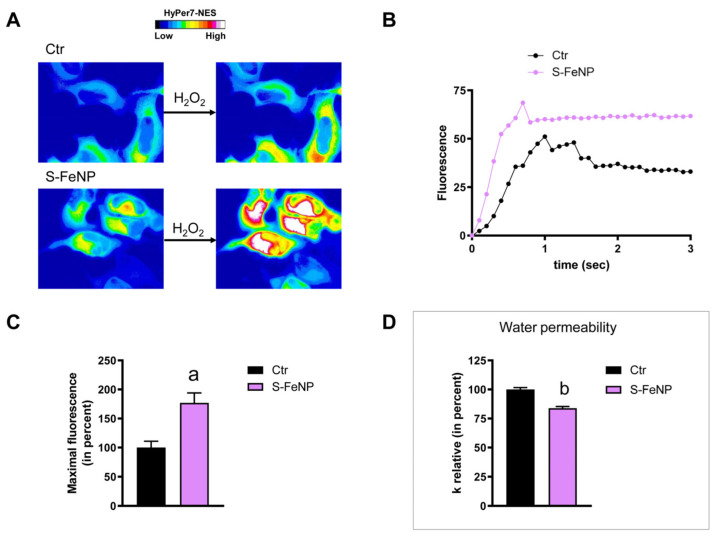
Hydrogen peroxide and water permeability in HeLa cells treated with S-FeNPs. H_2_O_2_ permeability in HeLa control cells (Ctr) and in HeLa cells treated for 2 h (S-FeNP) was evaluated after the addition of 50 μM H_2_O_2_. (**A**) Representative images extracted from videos showing the kinetics of H_2_O_2_ permeability in control and treated cells. The increased HyPer7-NES fluorescence is shown in pseudo color (upper panel; the scale used is indicated in the insert). (**B**) Representative time course of H_2_O_2_ fluorescence into control and treated cells. (**C**) Bars represent the maximal H_2_O_2_ fluorescence, which was obtained by computerized least squares regression, fitting the experimental points of the time courses of H_2_O_2_ transported curves with a one-phase exponential association equation (GraphPad Prism 4.00 2003). Maximal fluorescence values are means ± S.E.M. of 3 different experiments. a, *p* < 0.0005 (Student’s *t*-test). (**D**) Control cells (Ctr) and cells incubated with nanoparticles for 2 h (S-FeNP) were exposed to an osmotic gradient of 150 mOsm. Bars represent the osmotic water permeability of HeLa cells expressed as a percent of k relative. Values are means ± S.E.M. of 10–20 single shots for each of the 3 different experiments. b, *p* = 0.0176 (Student’s *t*-test).

**Figure 4:**
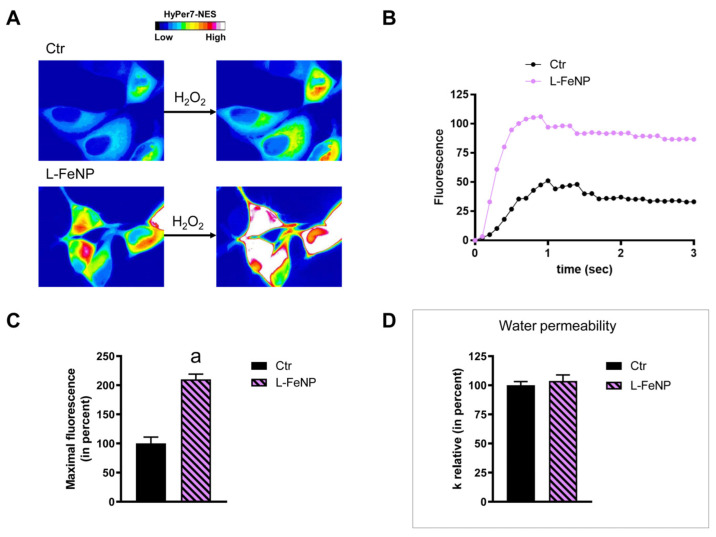
Hydrogen peroxide and water permeability in HeLa cells treated with L-FeNP. H_2_O_2_ permeability in HeLa control cells (Ctr) and HeLa cells treated for 2 h (L-FeNP) was evaluated after the addition of 50 μM H_2_O_2_. (**A**,**B**) See the legend of Figure 3. (**C**) Bars represent the maximal H_2_O_2_ fluorescence, which was obtained by computerized least squares regression, fitting the experimental points of the time courses of H_2_O_2_ transported curves with a one-phase exponential association equation (GraphPad Prism 4.00 2003). Maximal fluorescence values are means ± S.E.M. of 3 different experiments. a, *p* < 0.0001 (Student’s *t*-test). (**D**) Control cells (Ctr) and cells incubated with nanoparticles for 2 h (L-FeNP) were exposed to an osmotic gradient of 150 mOsm. Bars represent the osmotic water permeability of HeLa cells expressed as a percent of k relative. Values are means ± S.E.M. of 10–20 single shots for each of the 3 different experiments (Student’s *t*-test).

**Figure 5:**
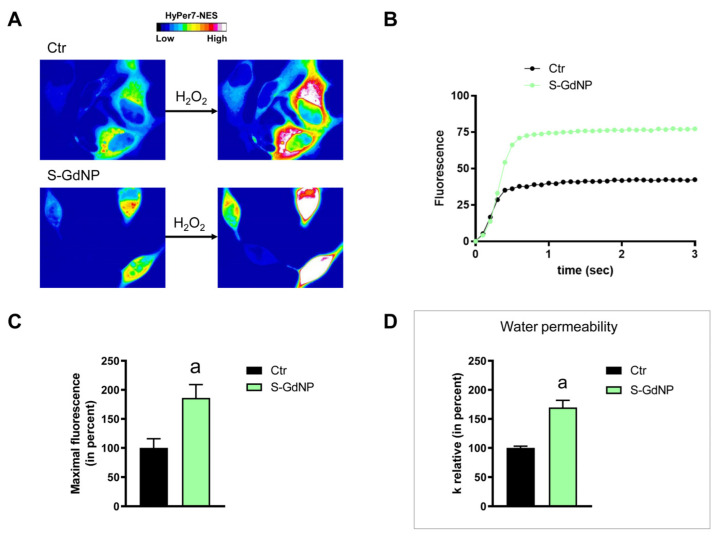
Hydrogen peroxide and water permeability in HeLa cells treated with S-GdNP. H_2_O_2_ permeability in HeLa control cells (Ctr) and HeLa cells treated for 2 h (S-GdNP) was evaluated after the addition of 50 μM H_2_O_2_. (**A**,**B**) See the legend of Figure 3. (**C**) Bars represent the maximal H_2_O_2_ fluorescence, which was obtained by computerized least squares regression, fitting the experimental points of the time courses of H_2_O_2_ transported curves with a one-phase exponential association equation (GraphPad Prism 4.00 2003). Maximal fluorescence values are means ± S.E.M. of the 3 different experiments. a, *p* < 0.0239 (Student’s *t*-test). (**D**) Control cells (Ctr) and cells incubated with nanoparticles for 2 h (S-GdNP) were exposed to an osmotic gradient of 150 mOsm. Bars represent the osmotic water permeability of HeLa cells expressed as a percent of k relative. Values are means ± SEM of 10–20 single shots for each of the 3 different experiments. a, *p* = 0.0317 (Student’s *t*-test).

**Figure 6:**
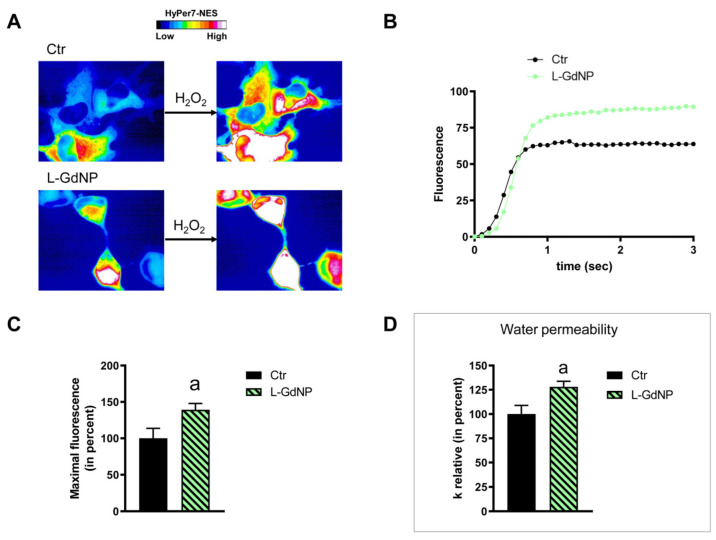
Hydrogen peroxide and water permeability in HeLa cells treated with L-GdNP. H_2_O_2_ permeability in HeLa control cells (Ctr) and HeLa cells treated for 2 h (L-GdNP) was evaluated after the addition of 50 μM H_2_O_2_. (**A**,**B**) See the legend of Figure 3. (**C**) Bars represent the maximal H_2_O_2_ fluorescence, which was obtained by computerized least squares regression, fitting the experimental points of the time courses of H_2_O_2_ transported curves with a one-phase exponential association equation (GraphPad Prism 4.00 2003). Maximal fluorescence values are means ± S.E.M. of the 3 different experiments. a, *p* = 0.045 (Student’s *t*-test). (**D**) Control cells (Ctr) and cells incubated with nanoparticles for 2 h (L-GdNP) were exposed to an osmotic gradient of 150 mOsm. Bars represent the osmotic water permeability of HeLa cells expressed as a percent of k relative. Values are means ± S.E.M. of 10–20 single shots for each of the 3 different experiments. a, *p* < 0.01 (Student’s *t*-test).

**Figure 7:**
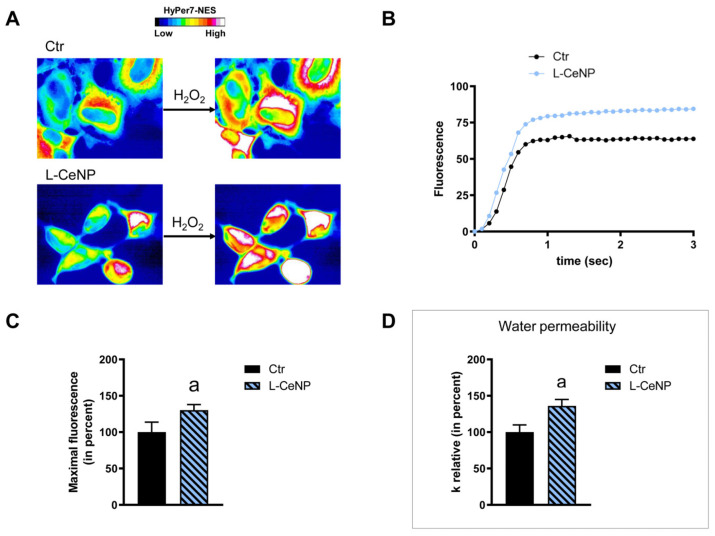
Hydrogen peroxide and water permeability in HeLa cells treated with L-CeNP. H_2_O_2_ permeability in HeLa control cells (Ctr) and HeLa cells treated for 2 h (L-CeNP) was evaluated after the addition of 50 μM H_2_O_2_. (**A**,**B**) See the legend of Figure 3. (**C**) Bars represent the maximal H_2_O_2_ fluorescence, which was obtained by computerized least squares regression, fitting the experimental points of the time courses of H_2_O_2_ transported curves with a one-phase exponential association equation (GraphPad Prism 4.00 2003). Maximal fluorescence values are means ± S.E.M. of the 3 different experiments (Student’s *t*-test). (**D**) Control cells (Ctr) and cells incubated with nanoparticles for 2 h (L-CeNP) were exposed to an osmotic gradient of 150 mOsm. Bars represent the osmotic water permeability of HeLa cells expressed as a percent of k relative. Values are means ± S.E.M. of 10–20 single shots for each of the 3 different experiments. a, *p* = 0.0147 (Student’s *t*-test).

**Figure 8:**
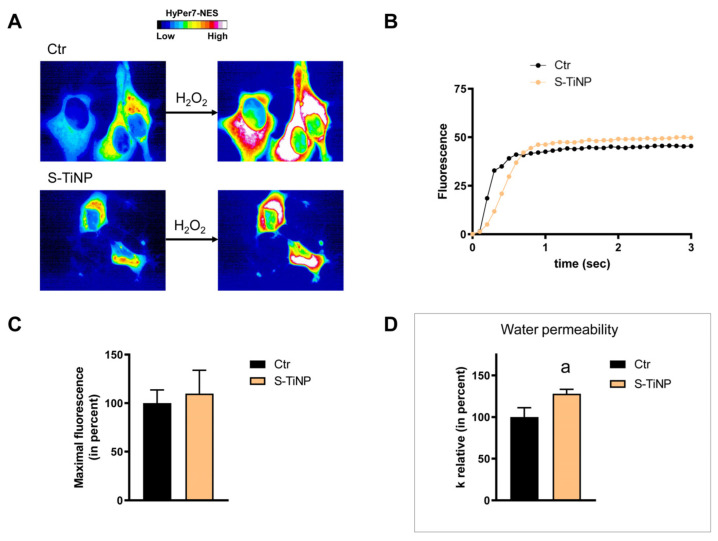
Hydrogen peroxide and water permeability in HeLa cells treated with TiNP. H_2_O_2_ permeability in HeLa control cells (Ctr) and HeLa cells treated for 2 h (TiNP) was evaluated after the addition of 50 μM H_2_O_2_. (**A**,**B**) See the legend of Figure 3. (**C**) Bars represent the maximal H_2_O_2_ fluorescence, which was obtained by computerized least squares regression, fitting the experimental points of the time courses of H_2_O_2_ transported curves with a one-phase exponential association equation (GraphPad Prism 4.00 2003). Maximal fluorescence values are means ± S.E.M. of the 3 different experiments (Student’s *t*-test). (**D**) Control cells (Ctr) and cells incubated with nanoparticles for 2 h (TiNP) were exposed to an osmotic gradient of 150 mOsm. Bars represent the osmotic water permeability of HeLa cells expressed as a percent of k relative. Values are means ± S.E.M. of 10–20 single shots for each of the 3 different experiments. a, *p* < 0.0001 (Student’s *t*-test).

HeLa cells were used to measure the time-course transport of H_2_O_2_ in the presence and in the absence of NP treatment. The effect of the NPs’ core composition, hydrodynamic size, and different surface functionalizations was considered. Concerning the effect of the hydrodynamic size of nanoparticles on H_2_O_2_ permeability, ANOVA followed by Dunnett’s *t*-test was conducted. No difference was observed between small and large NPs of Fe_3_O_4_, Gd_2_O_3_, and CeO_2_ (*p* > 0.05). Specifically, cells treated with S-FeNPs and L-FeNPs showed an increased transport of H_2_O_2_ by about 77%, and 110% respectively (Figure 3 and Figure 4). In the same way, S-GdNPs and L-GdNPs increased the H_2_O_2_ permeability by about 86% and 40%, respectively (Figure 5 and Figure 6). L-CeNPs increased the H_2_O_2_ permeability by about 40% (Figure 7), which is a value lower than the previously characterized S-CeNPs (with a 68% increase; Pellavio et al. [21]). TiNPs, on the other hand, showed no significant influence on H_2_O_2_ permeability (Figure 8). 

**Figure 9:**
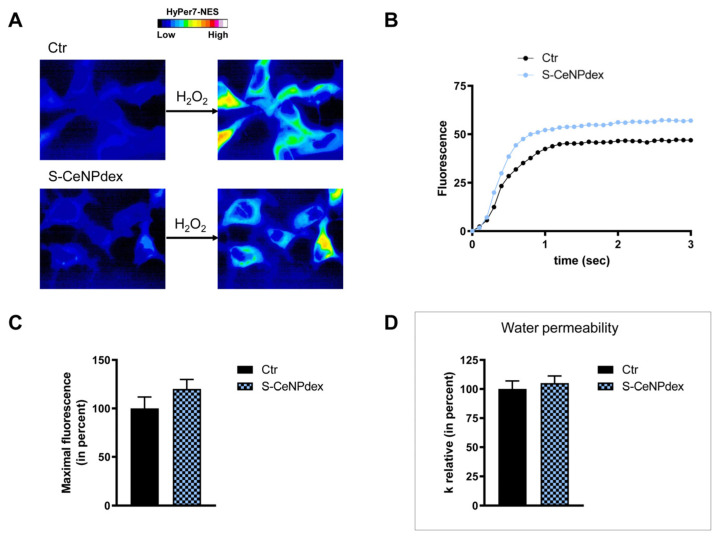
Hydrogen peroxide and water permeability in HeLa cells treated with S-CeNPdexs. H_2_O_2_ permeability in HeLa control cells (Ctr) and HeLa cells treated for 2 h (S-CeNPdex) was evaluated after the addition of 50 μM H_2_O_2_. (**A**,**B**) See the legend of Figure 3. (**C**) Bars represent the maximal H_2_O_2_ fluorescence, which was obtained by computerized least squares regression, fitting the experimental points of the time courses of H_2_O_2_ transported curves with a one-phase exponential association equation (GraphPad Prism 4.00 2003). Maximal fluorescence values are means ± S.E.M. of the 3 different experiments (Student’s *t*-test). (**D**) Control cells (Ctr) and cells incubated with nanoparticles for 2 h (S-CeNPdex) were exposed to an osmotic gradient of 150 mOsm. Bars represent the osmotic water permeability of HeLa cells expressed as a percent of k relative. Values are means ± S.E.M. of 10–20 single shots for each of the 3 different experiments (Student’s *t*-test).

Surface functionalization of nanoparticles, on the other hand, played a significant role in regulating the aquaporin’s behavior with respect to H_2_O_2_ transport. In the case of CeNPs, the substitution of a negatively charged capping agent, poly(acrylic) acid, with a neutral one, dextran, determined the loss of effect on H_2_O_2_ permeability (Figure 9).

### 2.4. The Effect of Nanoparticles on HeLa Cells’ Water Permeability

The effect of the NPs on water permeability was also investigated. HeLa cells were exposed to a 150 mOsm/L osmotic gradient in the presence and in the absence of NP treatment. The same NP parameters were considered. The size of the nanoparticles played a significant role in regulating water permeability, which was different from the observed effect on H_2_O_2_ permeability. On the other hand, it was difficult to identify the influence of the nanoparticles’ core composition. In fact, each type of nanoparticle showed an effect on water transport despite its nature, either inhibiting it (Fe_3_O_4_) or enhancing it (Gd_2_O_3_, CeO_2_, TiO_2_). Specifically, the treatment with S-FeNPs significantly decreased the water permeability by about 16% (Figure 3D), while the treatment with L-FeNPs did not change the permeability (Figure 4D). Both S-GdNP and L-GdNP treatments increased the water permeability by about 70% and 28%, respectively (Figure 5D and Figure 6D). Cells treated with L-CeNPs and with TiNPs showed an increase in water permeability by about 35% (Figure 7D and Figure 8D), while the previously studied S-CeNPs [21] increased water permeability by about 50%. Finally, the use of negatively charged PAA was proven to be essential for the activity of NPs on the AQP permeability, since the effect of CeNPs functionalized with dextran showed that CeNPsdex did not change the cell permeability to water (Figure 9D).

## 3. Discussion

Several metal (gold, silver, platinum, and palladium), non-metal (selenium), and metal oxide NPs have received growing attention for their properties in controlling the redox state of the cells with different mechanisms such as mimicking glutathione reductase, superoxide dismutase, and catalase activities [25,26]. Recently, we identified a new antioxidant mechanism for NPs that involves the modulation of peroxiporins’ scavenging properties [21]. Peroxiporins are AQPs that are able to facilitate the diffusion of H_2_O_2_, the most abundant ROS in the cells. The regulation of peroxiporins’ permeability can control the intracellular H_2_O_2_ concentration and thus the cell signaling pathway and the survival from oxidative stress [3,15]. Thus, by favoring the elimination of H_2_O_2_, peroxiporins work to protect the cell. CeNPs have been shown to increase both water and H_2_O_2_ permeability by interacting with AQP3, 6, and 8 [21]. This detoxifying effect was particularly evident during oxidative stress conditions. 

However, the mechanism responsible for the influence of NPs on aquaporins and peroxiporin is still unclear. In this work, we attempted to gain insight into this issue by altering the characteristics of the NP, in an attempt to evidence the property that has the strongest influence on the AQP gating regulation. However, a preliminary word of advice is essential in making such a comparison. Although we differentiated the produced NPs for their main chemical-physical characteristics, such as core composition, hydrodynamic dimension, functionalization, and surface charge, it must be realized that these characteristics might not be sufficient to describe the real NP structure when they interact with the biological system. The methods we used for the synthesis of the NPs do not allow strict control of the NPs’ morphology and dispersity. This is a common feature of the methods based on direct precipitation from aqueous solution. Methods allowing a much stricter control are based on non-aqueous solvents, but the resulting NPs cannot be directly used in a biological environment. As a result, similar hydrodynamic dimensions can be associated with different morphology, size distribution, and agglomeration levels in NPs deriving from different synthetic routes. It must also be considered that all the characterizations have been performed in an abiotic environment before the NPs are placed in contact with the biological medium. It is always difficult to evaluate the biological environment’s influence on the NPs’ structure and agglomeration level. Furthermore, it is well known that NPs tend to be surrounded by a protein corona, whose size, composition, and structure may vary considerably depending on a large number of parameters, including dimension, chemical nature, and surface charge. Such a corona represents the first layer of interaction between the NPs and the biological structures, and, in some cases, it is the main responsible for the biological activity of the nanostructures.

The main evidence from the analysis of our experimental results is that with only one exception, all NPs, independently from their chemical nature and size, produced an increase in water and H_2_O_2_ permeability. This is a remarkable result, as most of the chemical moieties influencing the AQP activity tend to reduce their permeability. The only exception is represented by S-FeNPs producing a reduction in the permeability of water. 

Within this general trend, some significant differences can be observed between the different NPs. The mechanism underlying the antioxidant activity of CeNPs is thought to be related to the equilibrium between the two oxidation states of cerium (Ce(III) and Ce(IV)) present on the surface of the CeNPs and that can easily switch from one to another [27,28]. To understand if the double oxidation state of the metal oxide core was an indispensable requirement to activate the AQP-mediated diffusion of H_2_O_2_, we treated HeLa cells with NPs whose metal constituents do not present an easy transition between different oxidation states, such as TiNPs and GdNPs. While GdNPs presented an activity comparable to that of CeNPs, TiNPs were ineffective. In this respect, no significant correlation can be identified. Regarding the anomalous behavior of TiO_2_, it must be noted that such NPs have been widely studied for their production of reactive oxygen species [29,30,31]. Moreover, their poor antioxidant activity has previously been reported [32,33].

The hydrodynamic size of the produced NPs, at least in the range used in this study, did not appear to change the diffusion of H_2_O_2_. Slightly higher permeability values were observed for smaller nanoparticles. This could be due to the increased uptake of smaller nanoparticles compared to bigger ones [34,35]. However, it is difficult to consider size-dependent effects, as nanoparticles in suspension can undergo aggregation in cell media and present a broader size distribution. When nanomaterials present a broader size distribution, smaller nanoparticles could inhibit the internalization of larger ones [36], and assessing which species is responsible for the permeability changes is challenging. 

The results obtained with NPs with different surface functionalizations suggest that the nature of the coating could play a more significant role in determining the AQP activity. In fact, the substitution of PAA with dextran induced a complete loss of function of the NPs, thus indicating that the negatively charged surface is essential for its role in modulating AQP permeability. PAA has been proposed to be an essential element in controlling the CeNP adhesion to cells [22]. In our previous work [22], we proposed that the PAA-coated CeNP interaction with the cell surface depends on the membrane components, specifically cholesterol, as the reduction of this component reduces the CeNP adhesion and its internalization. Although it is difficult to pinpoint how the PAA-NPs could promote an increase in the AQP-mediated diffusion of H_2_O_2_, it might be suggested that such interaction, which does not deplete the cell membrane from cholesterol, could alter the microenvironment around the AQP in a way to potentiate its detoxifying action. 

Considering the NP core components, the permeability experiments revealed another interesting feature of the NPs’ regulation of AQP functioning. While FeNPs and TiNPs differently regulated the permeability to hydrogen peroxide and water, CeNPs and GdNPs both increased. The interaction of the NPs with the AQPs probably modifies the aminoacid charges in the channel, altering the pore gating selectively. Similarly, acidic pH was shown to increase the glycerol but not the water permeability of AQP10 [37].

## 4. Materials and Methods

### 4.1. Nanoparticles Syntheses and Characterization

In this work, we used 50% poly(acrylic) acid (PAA), 99.99% pure Gd(NO_3_)_3_·H_2_O, 99.99% pure FeCl_3_·6H_2_O, and 99.99% pure FeCl_2_·4H_2_O, which were all acquired from Sigma Aldrich, Darmstadt, Germany; NH_4_OH (NH_3_ 28–30%) was acquired from Sigma Aldrich, Darmstadt, Germany; dH_2_O was sterilized by filtration with a 0.2 μm pore size filter (Minisart, Sartorious AG, Göttingen, Germany).

### 4.2. Synthesis of CeNPs (CeO_2_)

Small cerium oxide (CeO_2_) NPs were synthesized following a previously reported protocol [22]. To obtain larger NPs, the precipitating agent (NH_4_OH 30%) was added directly to the salt solution (rather than dropwise), and a lower mixing speed was used (500 rpm). Both these factors contribute to obtaining a broader size distribution. Specifically, 1.085g of Ce(NO_3_)3.6H_2_O was dissolved in 43 mL of distilled water, while 0.756 g PAA (50%) was dissolved in 31 mL of dH_2_O. Subsequently, 20 mL of PAA solution was added to the salt’s solution, together with 5 mL of NH_4_OH (28–30%). The suspension was then left under mixing at 30 °C for 48 h. At the end of the synthetic procedure, larger CeNPs (L-CeNPs) were washed and fractioned through centrifugation at different speeds. Specifically, the aqueous suspension of CeNPs was centrifuged at 1000, 10,000, and 17,000 rpm. Samples were characterized through Dynamic Light Scattering (DLS), and the fraction of interest (10,000–17,000 rpm) was identified and used for the biological studies. The resulting nanoparticles’ concentration was 6.6 mg/mL.

For S-CeNPdexs, 1.085g of Ce(NO_3_).6H2O was dissolved in 43.65 mL of distilled water. Concurrently, an equimolar solution of dextran (Streptococcus mutans, average mol wt 9000–11,000, Sigma Aldrich) was prepared in 20 mL of dH_2_O. The two solutions were mixed and stirred at 30 °C. After mixing, 5 mL of NH_4_OH (Sigma Aldrich, Darmstadt, Germany; 28.0–30.0%) was added drop by drop. The suspension was left under magnetic stirring at 30 °C for 24 h. At the end of the synthetic procedure, S-CeNPdexs were centrifuged and the supernatant was collected as the fraction of interest. The resulting nanoparticles’ concentration was 6.5 mg/mL. 

### 4.3. Synthesis of GdNPs (Gd_2_O_3_)

Both small (S-GdNP) and large (L-GdNPs) nanoparticles were obtained through the size selection of the same NP batch. Specifically, 0.5 g of Gd(NO_3_)_3_·6H_2_O was dissolved in 43 mL of dH_2_O, and PAA (50% wt) 0.05 M was prepared in 31 mL of dH_2_O. Subsequently, 20 mL of PAA solution was added to the salt solution under mild stirring, and precipitation was obtained by the dropwise addition of 5 mL of NH_4_OH (28–30%). The selection was carried out through centrifugation at 1000, 3000, and 17,000 rpm. S-GdNPs consist of the fraction isolated above 17,000 rpm, while L-GdNPs consist of the 3000–17,000 rpm fraction. The resulting nanoparticle’s concentration was equal to 6.4 mg/mL of Gd_2_O_3_. 

### 4.4. Synthesis of FeNPs (Fe_3_O_4_)

FeNPs were synthesized through a modification of the protocol proposed by Santra et al. [38]. Specifically, 0.36 g of FeCl_3_·6H_2_O and 0.2 g of FeCl_2_·4H_2_O were dissolved in 2.3 mL of HCl 0.4 M. Simultaneously, 0.93 g of PAA (50% wt) was dissolved in 5 mL of dH_2_O sterilized by Minisart sterile 0.22 µm filters. The salt solution was diluted in 12 mL of sterile dH_2_O, and the capping agent solution was subsequently added under mild stirring. Precipitation of FeNPs was obtained through the addition of 2.3 mL of NH_4_OH under higher speed stirring (630 rpm). The suspension was left under constant stirring at 30 °C for 1 h, and the produced NPs were later washed through centrifugation. To obtain different-sized nanomaterials, the clean suspension was then fractioned by centrifugation at different speeds: 1000–10,000, 10,000–17,000, and 17,000–100,000 g. S-FeNPs consist of the 17,000–100,000 g fraction, whereas L-FeNPs consist of the 10,000–17,000 g fraction. The Fe_3_O_4_ NP concentration in suspension was equal to 5.4 mg/mL. 

FeNPs were synthesized through a modification of the protocol proposed by Santra et al. [38]. Specifically, 0.36 g of FeCl_3_·6H_2_O and 0.2 g of FeCl_2_·4H_2_O were dissolved in 2.3 mL of HCl 0.4 M. Simultaneously, 0.93 g of PAA (50% wt) was dissolved in 5 mL of dH_2_O sterilized by Minisart sterile 0.22 µm filters. The salt solution was diluted in 12 mL of sterile dH_2_O, and the capping agent solution was subsequently added under mild stirring. Precipitation of FeNPs was obtained through the addition of 2.3 mL of NH_4_OH under higher speed stirring (630 rpm). The suspension was left under constant stirring at 30 °C for 1 h, and the produced NPs were later washed through centrifugation. To obtain different-sized nanomaterials, the clean suspension was then fractioned by centrifugation at different speeds: 1000–10,000, 10,000–17,000, and 17,000–100,000 g. S-FeNPs consist of the 17,000–100,000 g fraction, whereas L-FeNPs consist of the 10,000–17,000 g fraction. The Fe_3_O_4_ NP concentration in suspension was equal to 5.4 mg/mL. 

### 4.5. Functionalization of TiNPs (TiO_2_) 

Titanium oxide nanoparticle powders were bought from PlasmaChem GmbH (Rudower Chaussee, Germany; TiO_2_ nanopowder, 1–3 nm, rutile); 24 mg of TiO_2_ powders was suspended through sonication (20 min, 50 W) in sterile dH_2_O to obtain a 6 mg/mL concentration. A 6 mM solution of PAA was added. To enhance capping, 0.5mL of NH_4_OH 30% was added while stirring at 1200 rpm, and the suspension was kept under stirring at RT for 2 h 30 min. The suspension was then washed to remove excess PAA and ammonia through centrifugation and later fractioned. The only stable fraction resulting from this treatment consisted of the 10,000–17,000 g fraction. Successful functionalization was verified through Zeta Potential measurements. 

### 4.6. Nanoparticles Characterization 

NPs were characterized in terms of hydrodynamic size and surface charge. DLS was performed using a Nano ZS90 DLS analyzer (Malvern Instrument, Malvern, UK). The suspensions were diluted to about 1 mg/mL for the analysis. For each sample, 3 measurements of 11 runs were carried out. The same instruments were used to carry out the zeta potential measurements. Transmission Electron Microscopy (TEM) was also conducted using a JEOL TEM on suspensions diluted 1:20 in MilliQ water. Drops (2 µL) of suspension were placed on formvar/carbon-coated 250-mesh copper grids and left to dry for 10 min.

### 4.7. Cell Culture

HeLa cells were cultured in Dulbecco’s modified minimal essential medium–high glucose, supplemented with 10% fetal bovine serum, 1% L-glutamine, 1% penicillin, and streptomycin, and maintained at 37 °C in a humidified atmosphere of 5% CO_2_. For the NPs’ treatments, cells were washed and incubated with PBS containing NPs for 2 h.

### 4.8. Cell Viability

The cell viability of HeLa cells was evaluated after 2 h incubation with NPs diluted to 1:10, 1:20, 1:50, 1:100, and 1:200, using ReadyProbes^TM^ Cell Viability Imaging Kit (Blue/Green) (R37609, Thermo Fisher Scientific, Waltham, MA, USA). Briefly, cells were seeded at 50% confluency in 96-well black plates with clear bottoms. The next day, after the incubation with NPs, the medium was substituted with NucBlue^TM^ Live and NucGreen^TM^ Dead in PBS (both 2 drops/mL) and incubated for 30 min at room temperature (RT). After the incubation, the wells were washed three times with PBS. NucBlue^TM^ Live (Ex 360 nm, Em 460) and NucGreen^TM^ Dead (Ex 420 nm, Em 535 nm) fluorescences were measured using a CLARIOstar^®^ microplate reader (BMG LABTECH, Ortenberg, Germany). The viability was determined by calculating the blue signal vs. the green signal, and the ratio was normalized for total protein content [39].

### 4.9. Water Permeability Measurements

The stopped-flow light scattering technique was performed to evaluate the osmotic water permeability. Experiments were conducted at RT using an RX2000 stopped-flow apparatus (Applied Photophysics, Leatherhead, UK) with a pneumatic trigger accessory (DA.1, Applied Photophysics, Leatherhead, UK) coupled with the Varian Cary 50 spectrophotometer (Varian Australia Pty Ltd., Mulgrave, Australia). The intensity of the scattered light was measured at the wavelength of 450 nm with a dead time of 6 ms. Cells were exposed to a hypotonic gradient (150 mOsm/L), and then the cell swelling was measured for 60 s with an acquisition rate of one reading/0.0125 s. The initial rate constant k was calculated by fitting the experimental points of the time course of light scattering with a one-phase exponential decay equation (GraphPad Prism 4.00, 2003). Briefly, HeLa cells were scraped from the flasks, centrifuged, resuspended in PBS, and incubated with and without NPs. Two groups were considered: (1) untreated cells (control) and (2) cells treated for two hours with NPs.

### 4.10. Hydrogen Peroxide Indicator Transfection for Optical Imaging

The plasmid for the mammalian expression of cytoplasm-targeted ultrasensitive hydrogen peroxide indicator HyPer7 for optical imaging (pCS2+HyPer7-NES) was a generous gift from Vsevolod Belousov (IBCh, Moscow, Russia) (Addgene plasmid # 136467; http://n2t.net/addgene:136467 (accessed on 22 November 2023; RRID: Addgene_136467)) [40]. Furthermore, 60–70% confluent HeLa cells, seeded in 2 mL Petri dishes, were transfected with HyPer7-NES (1 μg DNA/dish) using the JetOPTIMUS DNA Transfection Reagent (# 117-15, Polyplus transfection, Illkirch-Graffenstaden, France) according to the manufacturer’s instructions. Firstly, the plasmid DNA (1 μg) was diluted in JetOPTIMUS Buffer (# 717-60, Polyplus transfection, Illkirch-Graffenstaden, France) and then combined with JetOPTIMUS Reagent in a 1:1 ratio (μg of DNA: μL of transfection reagent) and left at RT for 15 min. Meanwhile, the medium in Petri dishes was replaced by Opti-MEM, and after 15 min, the plasmid DNA solution was added dropwise to the cells. After 4 h at 37 °C, the Opti-MEM was removed, and a fresh complete medium was added. All the experiments were performed 24 h after transfection. 

### 4.11. Intracellular H_2_O_2_ Detection by HyPer7-NES Imaging

The effect of NPs on the H_2_O_2_ permeability Hyper7 oxidation was measured by a ratiometric method [40]. Confocal images were collected every 1–2 s for 1 to 5 min by dual excitation at 420 nm and 490 nm, and the emission was collected at 530 nm. Preliminary experiments showed that results obtained by ratiometric measurements were similar to those obtained by measuring the fluorescence of the HyPer7-NES biosensor excited at 490 nm and the emission collected at 530 nm. For this reason, the following method was routinely used. An Olympus BX41 microscope with a 60× water immersion objective (LUMPlanFI 60×/0.90 w, Olympus Italia, Segrate, Italy) was used to visualize the fluorescence of transfected cells. HyPer7-NES transfected cells were pretreated for two hours with the NPs, washed with a physiological buffer (140 mM NaCl, 5 mM KCl, 2 mM CaCl_2_, 1 mM MgCl_2_, 10 mM D-glucose, and 1 mM HEPES, pH 7.4), and incubated for 10 min at RT with the same buffer. Images were acquired using a CCD camera (DMK 33UP1300, The Imaging Source Europe GmbH, Bremen, Germany) and collected at 10 fps by IC capture software (version 2.5, Imaging Sourse; accessed date 26 December 2023). H_2_O_2_ was added to the cells at a final concentration of 50 μM. Image processing was performed with ImageJ, Rasband, W.S., ImageJ, versione 1.8.0 U. S. National Institutes of Health, Bethesda, Maryland, USA, https://imagej.net/ij/, accessed on 27 December 2023.

### 4.12. Statistics

All data were expressed as means ± S.E.M. (Standard Error Mean). The significance of the differences between the means was evaluated by using a one-way ANOVA, followed by Dunnett’s multiple comparison test, or Student’s *t*-test. All statistical tests were carried out with GraphPad Prism 4.00, 2003.

## 5. Conclusions

**Figure 10:**
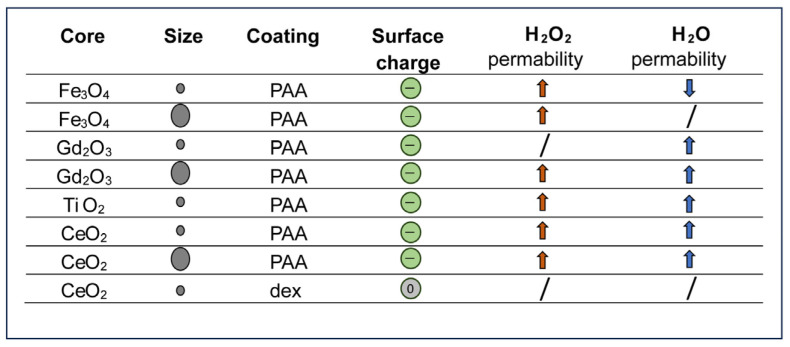
Summary of the NPs’ characteristics and permeability changes. Gray circles indicate small and large NPs. Green circles indicate the negative, while the gray indicates the neutral functionalization. Red arrows indicate H_2_O_2_ permeability, while blue arrows indicate H_2_O permeability. Arrows pointing up or down indicate increased or decreased permeability. PAA, poly(acrylic) acid; dex, dextran.

The results presented here, and summarized in Figure 10, suggest that NPs can strongly influence AQP activity. NPs functionalized with PAA produce a significant increase in both water and H_2_O_2_ permeability independently from their size and core chemical nature. This is a remarkable result as an enhancement in AQP permeability has seldom been reported. Although differences in hydrodynamic size and chemical nature produce differences in the extent of such influence, no significant trends have been identified. TiO_2_ NPs have represented the only exception. Such a compound, however, is well known for being a photoactive material, presenting a strong tendency to produce free radicals and reactive oxygen species. 

The type of surface functionalization presented a much stronger influence on the NPs’ activity. In fact, if the strongly ionic PAA functionalization is replaced with mildly ionic dextran, the AQP activity of the NPs is completely lost. This effect could be related to the difference in ionic charge, which might interfere with the ionic distribution in the AQP channel, or to the ability of PAA to interact with some cell membrane components, particularly cholesterol. 

The evidence we reported is preliminary in nature, and further studies are required in order to better clarify the mechanism responsible for this unusual and unexpected influence of NPs on AQP activity. However, the possibility of modulating the AQP permeability considerably suggests that such nanostructures can play a significant role in controlling oxidative stress in several pathologies associated with an increased oxidative status. 

## Data Availability

Data are contained within the article.
